# Experiment and numerical analysis of prestressed unequal-walled rectangular concrete-filled steel box beams

**DOI:** 10.1038/s41598-024-84473-2

**Published:** 2025-01-02

**Authors:** Qi Su, Guangyuan Fu, Jian Yang, Siping Li

**Affiliations:** School of Naval Architecture Ocean & Civil Engineering, Shanghai Jiao Tong University, Shanghai, 200240 China

**Keywords:** Prestressed, Partially filled, Steel box-concrete, Static test, Bending performance, Civil engineering, Mechanical engineering

## Abstract

When long-span beams undergo large, the strength of the beam material cannot be fully utilized. To solve this problem, a prestressed unequal-walled rectangular concrete-filled steel box (PURCFSB) beam is proposed in this paper. The prestressing is added to the concrete-filled steel tubular (CFST) beam and the section is designed. This beam is an improved concrete-filled steel tubular (CFST) beam with flanges of unequal thickness; the upper part of the steel box contains concrete, and the lower part is fitted with prestressed steel bars. Bending tests on ten PURCFSB beams with different concrete filling ratios and prestress levels revealed that prestressing can increase the yield bending moment and delay the cracking of concrete. The appropriate filling ratio can improve the structure’s yield moment and ultimate bearing capacity and reduce its self-weight while avoiding concrete cracking within the scope of work. Finite element analysis of the whole bending process of PURCFSB beams, carried out using ABAQUS software, yields results that are in good agreement with the experimental results. Parametric analysis (based on the validated finite element model) of the effects of the filling ratio and prestress level on the bending performance revealed that PURCFSB beams have good bearing capacity and deformation performance.

## Introduction

Steel–concrete composite structures are widely used because they combine the advantages of both materials^1–4^. With the emergence of large-span and heavy-load structures, prestressing technology (which reduces structural weight and improves structural fatigue resistance) has also developed rapidly^5–10^and has been applied to steel–concrete composites.

Several urgent engineering problems involving steel–concrete structures still await further excavating the bearing capacity of the components, improving the strength and bending stiffness of long-span beams during normal use, and reducing component weight^11–13^. To solve these problems, new sectioned forms of composite structures have been proposed. Li et al. suggested the use of rectangular concrete-filled steel tubes (CFSTs) with walls of unequal thickness^14–16^. These structures (including bending beams, long columns under compression, and short columns under eccentric compression) have been experimentally and theoretically shown to have good ductility and bearing capacity. When the steel ratio is 0.18, the bearing capacity of a rectangular CFST with unequal wall thickness is 23.4–35.0% greater than that of a CFST with equal wall thickness. Zhong et al. proposed a sectioned steel-box-concrete composite beam, dividing the steel box into upper and lower parts, with concrete filling the upper part. Under the same bending capacity, the weight of such beams is 59.5% less than that of ordinary steel–concrete composite beams^17–20^. Moreover, the ultimate bending strength of steel-box-concrete beams is 40% greater than that of steel-box beams, and the ductility is increased approximately eightfold^21^. The influence of the concrete filling ratio has been less studied, although a numerical simulation was carried out in^17^.

The use of prestressed CFSTs has been proposed as a means to avoid the decrease in structural stiffness caused by concrete cracking. Zhan et al. carried out bending tests on eight large-Sects. (300–450 mm) prestressed CFST beams and reported that the cracking moment increased by approximately 400%^22^. Ghaemdoust et al. studied the structural characteristics of prestressed concrete-filled steel tubular flange beams (CFSTFBs) under bending; compared with I-beams with flat flanges, CFSTFBs had greater bending and shear strengths^23^. Ren et al. also studied the bending behavior of multi-prestressed deep beams with pretensioning and posttensioning under concentrated force^24^.

Most existing studies of prestressed CFSTs have focused on the compression performance of columns; however, few studies have investigated the bending of prestressed CFST beams^25–27^. The existing studies also use numerical simulations to analyse the different reinforcement methods of prestressed tendons, the types and quantities of prestressed tendons, and the influence of eccentricity, whereas there are few experimental studies of different prestress levels exist^28–31^.

To help fill these gaps in the literature and realize the application potential of prestress, a composite sectioned form of a prestressed unequal-walled rectangular concrete-filled steel box (PURCFSB) is proposed in this study. Compared to references^17] and [18^, this study has incorporated prestressed reinforcement into the cross-section and varied the concrete filling ratio. The thicknesses of the upper and lower flange plates are not equal. The upper part of the steel box is filled with concrete, and the lower part is equipped with external prestressed steel bars. Through experiments and finite element numerical simulations, the influence of the concrete filling ratio and prestress level on the flexural performance of the PURCFSB beam is discussed, providing a reference for this structure in engineering applications.

## Experimental overview

### Experimental design

**Fig. 1:**
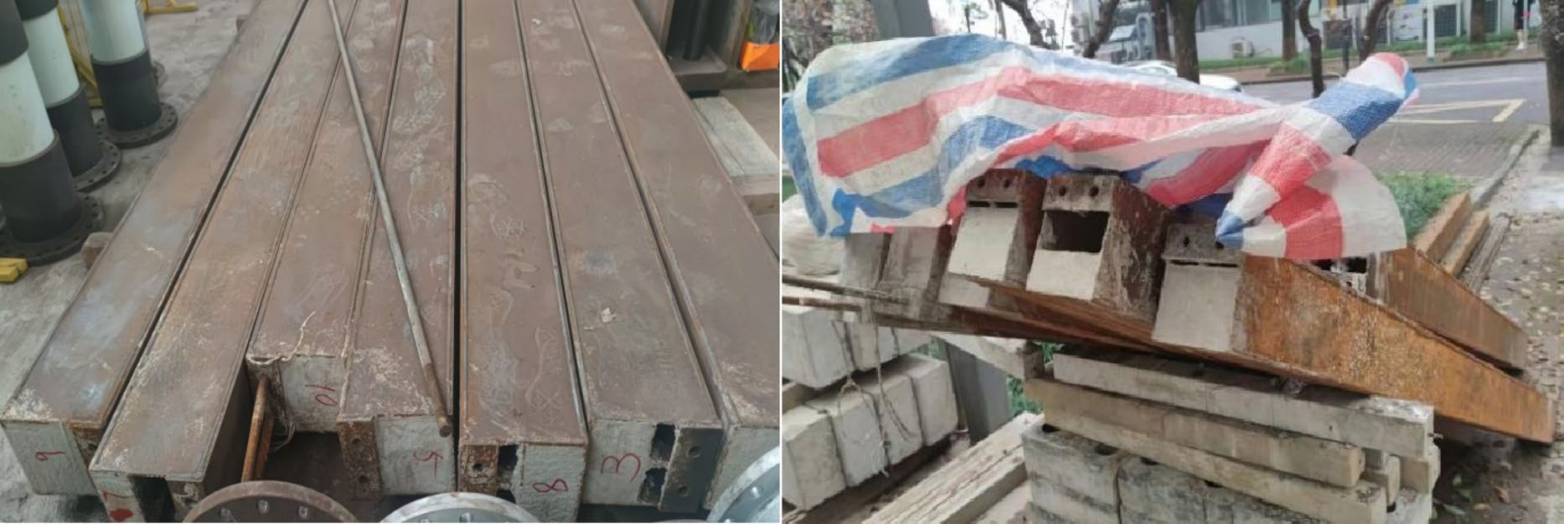
Concrete–filled rectangular steel Tube beam specimens.

**Fig. 2:**
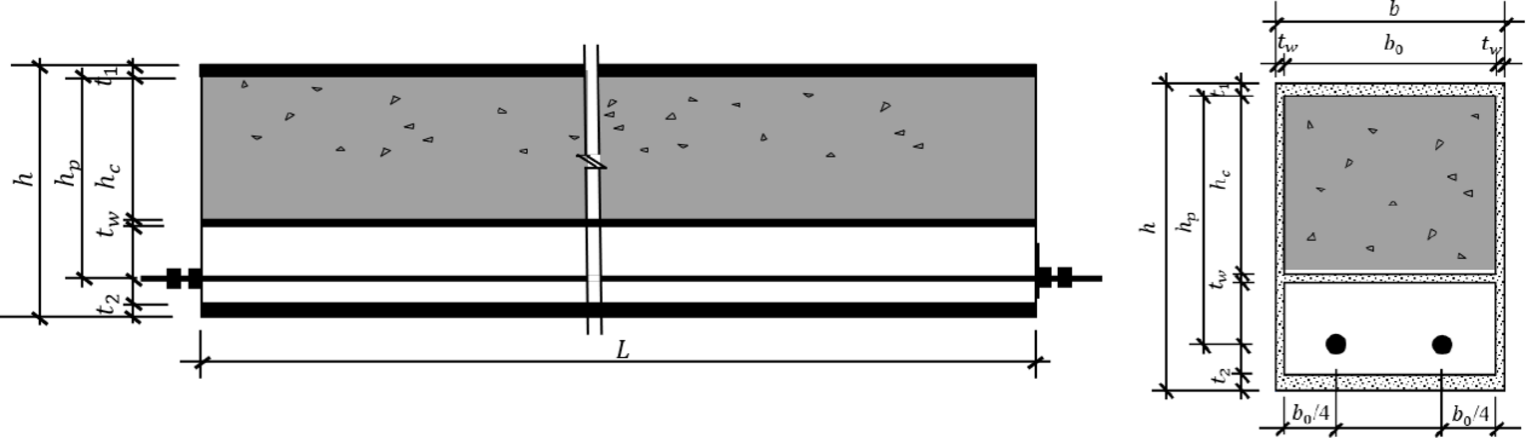
PURCFSB beam specimen section diagram.

The specimens consisted of 10 beams, divided into two groups according to the prestress level and different concrete filling ratios (0, 1/3, 1/2, 2/3, and 1). The upper part of the steel box was filled with concrete. The outer contour sizes of all the specimens and the thicknesses of the corresponding steel plates were the same. The specific size parameters and the cross-sectional forms of the specimens are shown in Table 1; Figs. 1 and 2. *h*_*p*_=162 mm, where *h*_*p*_ represents the distance between the prestressed steel bars and the bottom of the upper flange plate. The position of the intermediate diaphragm is determined by the concrete filling rate; there is no intermediate diaphragm when the concrete filling rate is 0 or 1.

The prestressed steel bars BR3-D, BR4-B, and BR4-E used two round (18 mm in diameter) #45 steel bars; the other specimens used two round (18 mm in diameter) Q235 steel bars. The steel plates were made of Q235 steel, and the grade of the concrete was C50. All the specimens were measured according to the methods specified in the relevant standards^32,33^, The average elastic modulus of steel and steel plate was 200 GPa. The strength of the concrete was measured for a cubic test block with a side length of 150 mm, formed and cured under the same conditions as the specimens: it was cured for 28 days, then placed in outdoor ventilation. The elastic modulus of the concrete was 34.6 GPa. All the steel and concrete strength statistics are shown in Table 1.

**Table 1:** List of test specimens. BR represents the bending beam, 1–5 represents the concrete filling ratios from small to large 0, 1/3, 1/2, 2/3, 1, A–E represents the prestress levels from small to large 0, 128, 192, 240, and 288 MPa.

Specimen	t_1_ (mm)	t_2_ (mm)	t_w1_ (mm)		t_w2_ (mm)	L (mm)	b (mm)	h (mm)	f_1_ (MPa)	f_2_ (MPa)	f_w_ (MPa)	f_py_ (MPa)	f_pu_ (MPa)	f_cu_ (MPa)
BR1-B	7.64	10	4.88	4.8	/	3000	198.8	151.8	301.2	285.1	309.3	288.3	424.9	/
BR2-C	7.68	9.43	5.06	4.95	4.9	3000	200.8	150				288.3	424.9	48.5
BR3-B	7.75	10.23	4.81	4.69	4.81	2999	199.4	150.6				288.3	424.9	51.1
BR4-B	7.66	10.12	4.84	4.92	4.74	2997	199	150.4				362.5	605.8	61.6
BR5-C	7.66	9.94	4.82	4.81	/	2997	201.2	150.4				288.3	424.9	51.1
BR1-C	7.65	10.04	4.85	4.86	/	2999	199.4	150.6				288.3	424.9	/
BR2-D	7.71	9.66	4.90	4.82	4.95	2995	199.4	150				288.3	424.9	48.5
BR3-D	7.77	9.46	4.84	4.91	4.75	2998	201.2	149				362.5	605.8	51.6
BR4-E	7.65	10.05	4.77	4.85	4.73	2997	199.8	149.2				362.5	605.8	51.6
BR5-A	7.7	10.03	4.75	4.88	/	2999	199.4	150				288.3	424.9	51.1
average	7.69	9.90	4.85	4.85	4.81	2998	199.84	150.2	/	/	/	/		51.9

### Experimental process

**Fig. 3:**
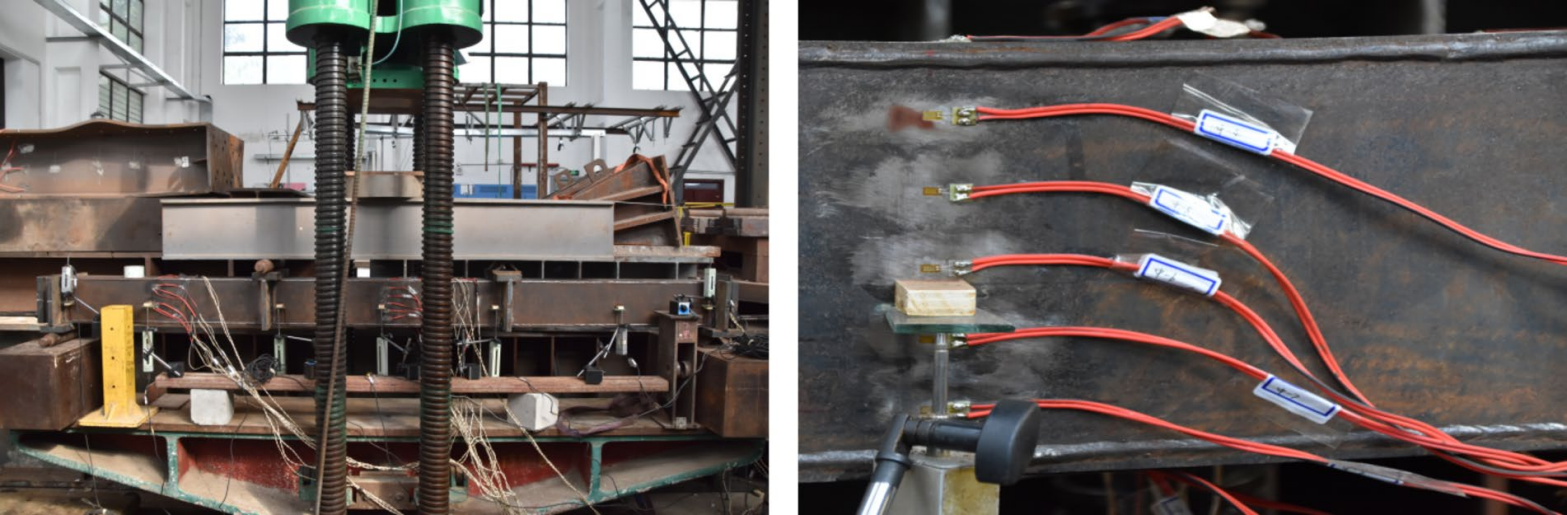
Loading device.

**Fig. 4:**
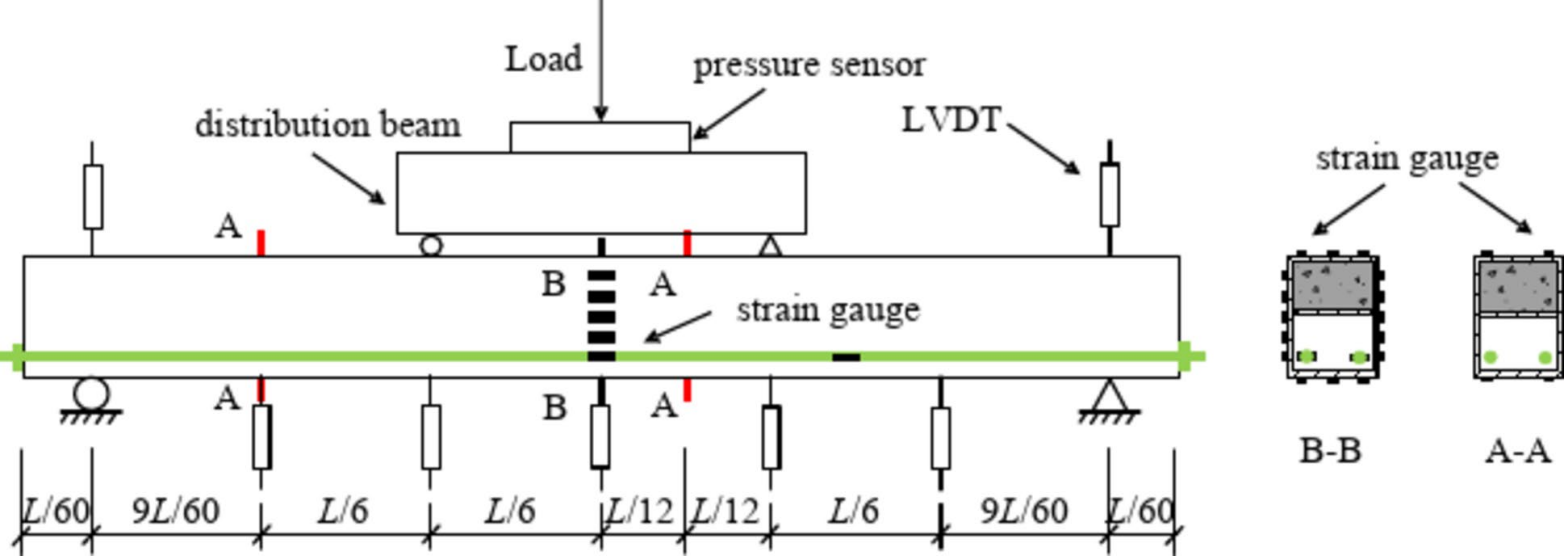
Schematic diagram of strain gauge arrangement.

The test was carried out in the structural laboratory of Shanghai Jiaotong University. The process is shown in Figs. 3 and 4.

The total length *L* of each specimen was 3000 mm; the effective length *L*_0_ was 2900 mm, as both ends were simply supported. The test process was divided into two stages: the prestressed stage and the bending stage. The prestressed loading method involved one-end tension, and the anchoring method involved a high-strength nut. In the bending stage, the load was applied by a 2000 kN hydraulic jack, with four-point bending used as the loading method. The concentrated load was evenly distributed to the component by the distribution beam, and the length of the pure-bending section was 1000 mm. The load value was measured with a pressure sensor, the deflection of the beam was measured with a linear differential variable transformer (LVDT), and strain gauges were used to measure the strain at different positions of the prestressed steel bars and beams. The positions of the pressure sensor, strain gauge, and LVDT are shown in Fig. 4. The pressure sensor was located in the middle above the distribution beam. The strain-gauge position on the steel box is shown in Section A-A and B-B. There are two Sections A-A ; the left Section A-A is 1000 mm away from the center, used for detecting the strain in the bending-shear segment, and the right Section A-A is 250 mm away from the center, used for verifying the strain in the pure bending segment at Section B-B. The strain gauges for the steel bar were located at mid-span and 750 mm from mid-span. The data were collected automatically and continuously by a DH3816 static-strain test system. The entire loading process lasted approximately 2.5 h.

## Experimental results

### Experimental phenomena and deformation characteristics

The test termination condition was that the deflection of the applied-load point reached approximately *L*/30. The unequal-walled steel-box beams BR1-B, BR1-C, BR2-C, and BR2-D experienced local buckling in the later stage of loading. All specimens had a ratio of displacement at the ultimate bending moment to displacement at the yield bending moment of approximately 2.4 to 3.75. Additionally, after reaching the displacement corresponding to the ultimate bending moment, the load-carrying capacity of the specimens did not decrease until the end of the test at a displacement of 100 mm, except that the loads of BR2-C and BR2-D decreased because of local buckling in the later stage of loading. These findings indicate that the PURCFSB beams had good ductility.

**Fig. 5:**
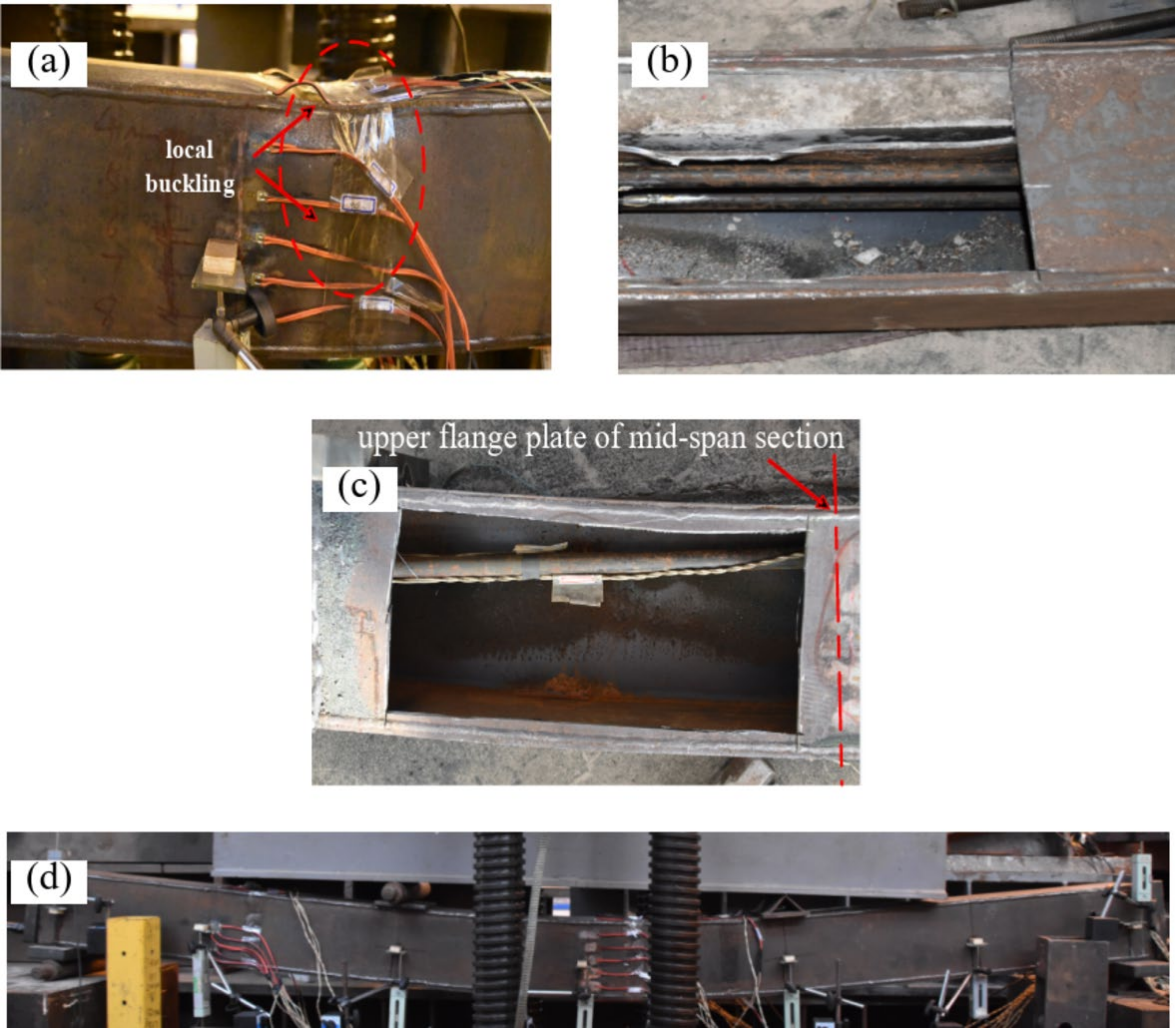
The final form of the beam. **(a)** Local buckling in mid-span of BR1-B; **(b)**Delamination of concrete of BR2-C; **(c)** The relative position change of prestressed steel bar in BR1-C; **(d)** Deformation of BR2-D.

Figure 5 shows the final form of some specimens at the end of the test. BR1-B, BR1-C, BR2-C, and BR2-D exhibited local buckling at the later stage of loading, as shown in Fig. 5 (a), and their bearing capacity decreased. When the tests were over, BR2-C and BR2-D were cut open as shown in Fig. 5 (b). Examinations revealed that delamination had occurred between the concrete and the steel-box clapboard, leaving the concrete essentially intact; this indicates that the concrete beam was kept under compression during the bending process when the concrete filling ratio was equal to 1/3. Specimens BR3-B, BR4-B, BR5-C, BR3-D, BR4-E, and BR5-A exhibited the sound of concrete cracking during the testing process, and no local buckling was observed by the end of the loading. As shown in Fig. 5 (c), in the mid-span of specimen BR1-B, the position of the steel bar was close to the upper flange. This finding suggests that in the later stages of loading, for beams with a lower concrete fill ratio, the rebar at the mid-span section may shift from below to above the neutral axis, which can induce negative moments due to prestress, adversely affecting the structural capacity. Although this situation occurs in the later stage of loading, far beyond the normal use range of the specimen, the influence of secondary effects must be considered for components with large spans. Therefore, a steering block should be set to limit the vertical displacement of prestressed steel bars in engineering applications.

**Fig. 6:**
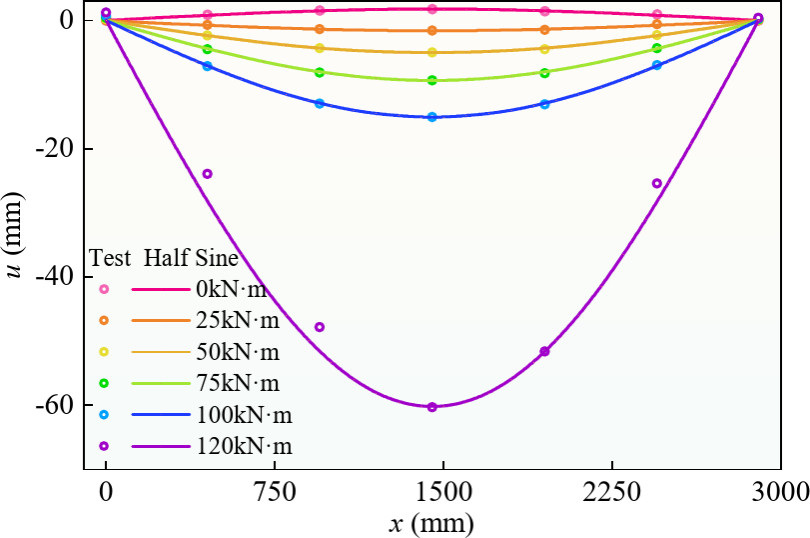
Deflection distribution curve of BR3-D.

The deflection distribution curve of the whole process for specimen BR2-D is shown in Fig. 6; the abscissa represents the distance from one end of the beam along the long direction of the beam. The other specimens had curves similar to those of BR2-D. The deflection‒distribution curve of the beam conformed to the sine‒half‒wave hypothesis. Under the action of prestress, the initial state of the beam was an inverted arch.

### Moment‒strain curve

**Fig. 7:**
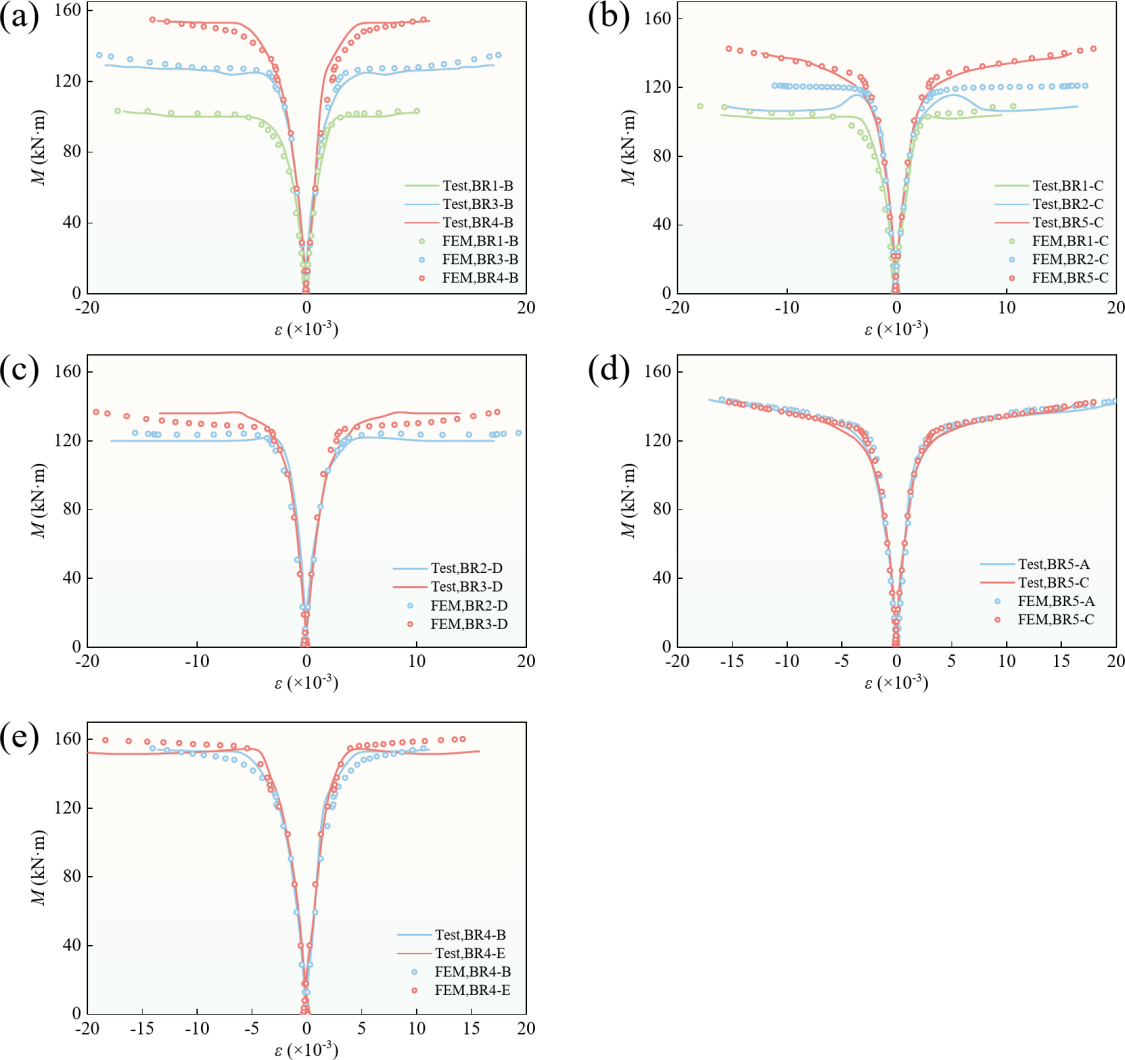
Moment–strain curve. **(a)**,** (b)** and **(c)** are comparisons between beams with different fill rates. **(d)** and **(e)** are comparisons between beams with different degrees of prestressing.

The relationship between the strain and bending moment of the upper and lower flanges of the mid-span section is shown in Fig. 7. Before the steel tube yielded, the strains of the upper and lower flanges increased linearly. As the bending moment increased, the upper and lower flanges successively yielded. After the steel box yielded, the slope of the curve decreased rapidly until the bending moment approached its ultimate value, where the slope was close to zero.

Table 2 lists the concrete crack moment, upper-flange and lower-flange yield moments, and ultimate moment. When the tensile edge strain of the beam reached 0.01 and there was no load‒drop section, the bending moment was the ultimate bending moment. When there was a load-drop section, the maximum bending moment before the strain reached 0.01 was the ultimate bending moment^34^. According to Table 2, the yield moments of the upper and lower flanges of BR3-B and BR3-D were similar. When the filling ratio was between 1/3 and 1/2, the lower flange yielded first; otherwise, the upper flange yielded first. As shown in Fig. 7, the upper- and lower-flange strains of all the specimens were almost symmetrical about the longitudinal axis at the beginning of loading, and the strain increased linearly with the bending moment. As the upper and lower flanges of the steel box yielded, the load growth rate of the specimen decreased rapidly, and the bending moments of all the specimens remained essentially unchanged after the strain at the edge of the steel box reached 0.01.

Beams with different filling ratios are shown in Fig. 7(a), (b)and (c). A larger filling ratio corresponded to a slower decrease in the bearing-capacity growth ratio of the later specimens, indicating that the stiffness decreased slowly. According to Fig. 7(d) and (e), because the beams had the same cross-sectional shape, the beams had little effect on the geometric stiffness at small deformations with different prestressing levels, and different prestress levels had little effect on the strain of the upper and lower flanges during the whole bending process of the structure.

**Table 2:** Yield moment and ultimate moment of specimen. The value of the subscripted FEM is the result of the finite element model. The value of the subscripted TEST is the result of test.

Specimen (KN·m)	M_cr, Test_	M_cr, FEM_	M_cr, FEM_/ M_cr, Test_	M_y1_	M_y2_	M_y1_/ M_y2_	M_y, FEM_	M_y, FEM_/ M_y, Test_	M_u, Test_	M_u, FEM_	M_u, FEM_/ M_u, Test_
BR1-B	/	/	/	77	85	0.906	74.0	0.961	103	103.1	1.001
BR1-C	/	/	/	80	92	0.870	76.8	0.96	104	105.2	1.012
BR2-C	/	/	/	102	93	1.097	96.9	1.042	118	120.3	1.019
BR2-D	/	/	/	104	97	1.072	103.2	1.064	122	123.1	1.009
BR3-B	109	108.7	0.997	100	96	1.042	101.4	1.056	127	127.6	1.005
BR3-D	112	111.2	0.993	105	100	1.050	107.2	1.072	137	130.3	0.951
BR4-B	48	45.1	0.940	104	117	0.889	102.9	0.989	153	148.1	0.968
BR4-E	68	71.1	1.046	106	121	0.876	103.9	0.98	155	151.2	0.975
BR5-A	12	11.6	0.967	94	108	0.870	99.6	1.06	134	134.9	1.007
BR5-C	25	23.3	0.932	104	110	0.945	103.2	0.992	134	134.5	1.004
Average			0.979					1.018			0.995
Standard deviation			0.042					0.045			0.022

### Moment–deflection curve

**Fig. 8:**
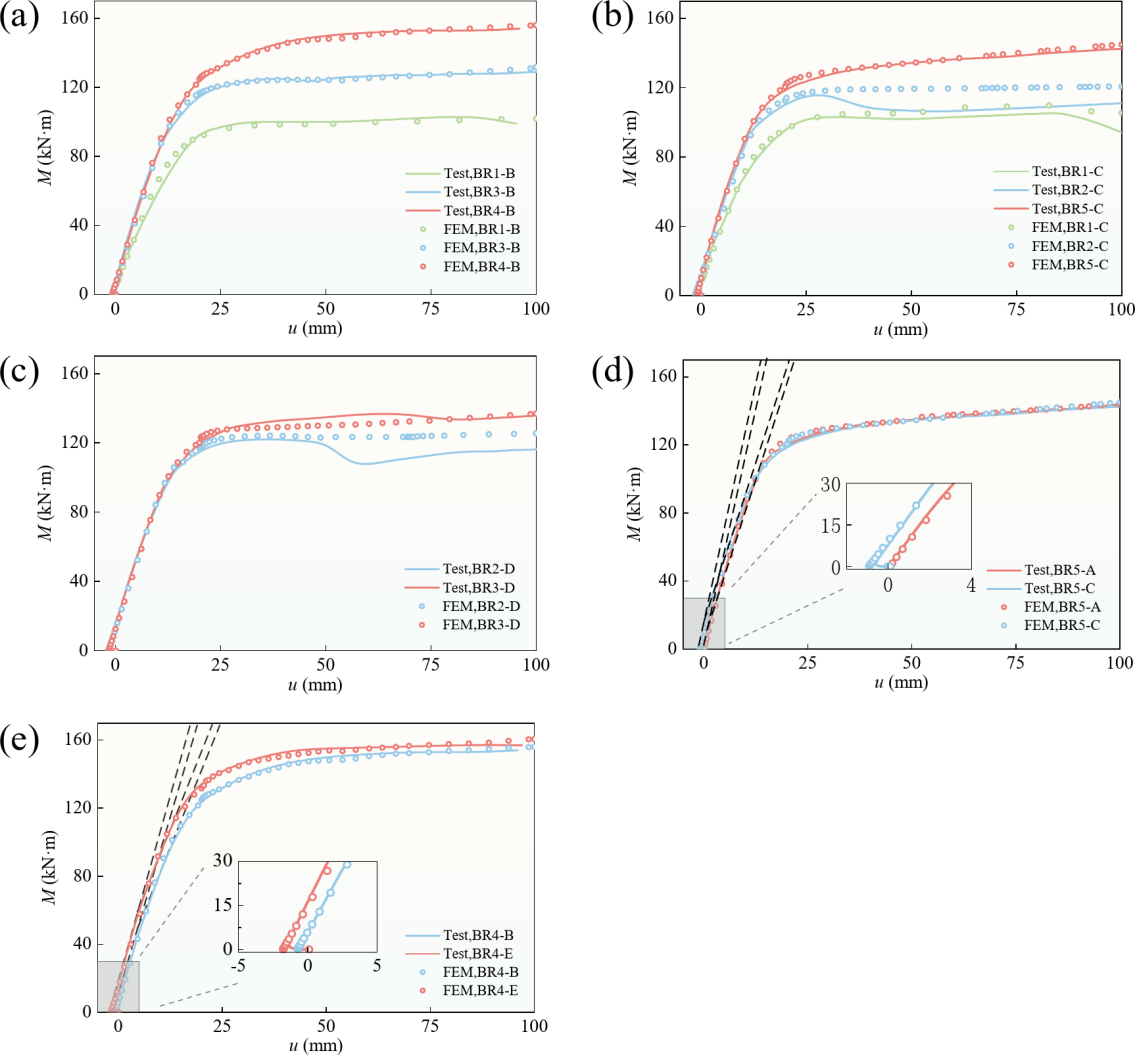
Load–deflection curve. **(a)**,** (b)** and **(c)** are comparisons between beams with different fill rates. **(d)** and **(e)** are comparisons between beams with different degrees of prestressing.

The mid-span bending moment*–*deflection curve of the test beam reflected the overall working performance of the flexural member. As shown in Fig. 8, it can be roughly divided into elastic and plastic stages. From the start of loading until the upper or lower flange plate of the steel box yielded, the test beam exhibited elastic deformation properties, the bending moment–deflection curve was approximately straight, the stiffness was mostly unchanged, and the tensile stress of the prestressed steel bar increased. According to Table 2, when the filling ratio was 1 or 2/3, the concrete cracked in the elastic stage, Referring to Fig. 8 (d) and (e), after reaching the cracking moment, the curve continued to increase linearly, but with a reduced slope, indicating that the structural stiffness decreased, yet it was still in the elastic stage. In the plastic stage, the bending moment–deflection curve had an inflection point until a plastic hinge appeared. After the plastic hinge appeared, the bending moment essentially remained unchanged as the deflection of the beam increased, reflecting that the beam has good ductility. In Fig. 8 (a), (b), and (c), BR2-C and BR2-D show obvious load declines in the later stage of loading; these decreases were caused by local buckling. BR1-B and BR1-C also experienced local buckling, but the bearing capacity did not decrease significantly; this indicates that local buckling caused the partially filled concrete to lose the steel-tube constraint. Thus, local buckling has a greater influence on a steel-box-concrete beam than on a steel-box beam does, and designers of steel-box-concrete beams should ensure that the filling ratio is not too small. At the same time, the numerical simulation curves did not exhibit a decline, which is due to the absence of local initial defects in the simulation. Our model primarily focuses on the nonlinear behavior of materials and the geometric nonlinearity of structures, which are the dominant factors in the experiment. Therefore, the discrepancies caused by local initial defects do not affect the theoretical analysis of structural load-bearing capacity.

A comparison of (a), (b), (d), and (e) in Fig. 8 reveals that the influence of the filling ratio on the flexural performance of beams was greater than that of the prestress level. Figure 8 (c) shows that the bearing capacity of the structure was not much different when the filling ratio was 1/2 rather than 1/3, but the structure was safer and had better ductility. A comparison of (d) and (e) in Fig. 8 reveals that the influence of the high yield strength of the steel bar on the beam was greater than that of the ordinary steel bar. According to Table 2; Fig. 8, the appropriate filling ratio and prestress level increased the cracking moment of the specimen by 108.3%. Compared with a prestressed unequal-walled steel-box beam, the yield moment increased by 37.7% and the ultimate moment increased by 50.5%; compared with an unequal-walled CFST beam, the yield moment increased by 12.8% and the ultimate moment increased by 15.7%.

### Mid-span section strain analysis

**Fig. 9:**
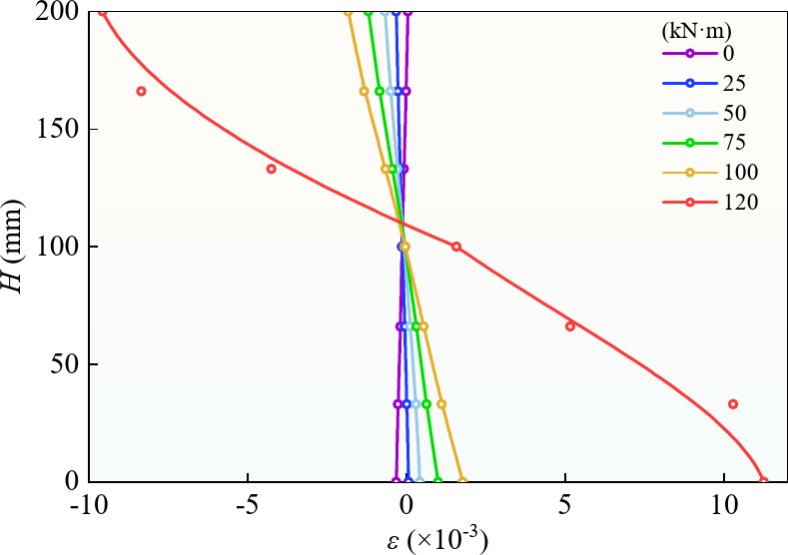
Strain analysis of cross section.

Figure 9 shows the strain of the mid-span section of the prestressed steel box-concrete beam along the beam height under different displacement loads. (the figure shows specimen BR3-D; the other specimens yielded similar results.) As shown in Fig. 9 that the cross-sectional strain changed linearly along the beam height under the bending moment of each characteristic point. This indicates that the section deformation of the prestressed steel-box girder was maintained as a plane, conforming to the plane-section assumption, not only in the elastic stage but even after some sections had entered the plastic stage. However, because of local buckling, the deformation of the empty steel box did not conform to the plane-section assumption after reaching the ultimate load.

## Finite element numerical analysis

In addition to the experiments described in Sect. 3, a numerical model of the PURCFSB beam using the finite element method (FEM) was developed. This section briefly describes the model and how well its results match the experimental results. The verified model was then used to determine the effects of varying the filling ratio and prestress, as described in Sect. 5.

### Material constitutive model

**Fig. 10:**
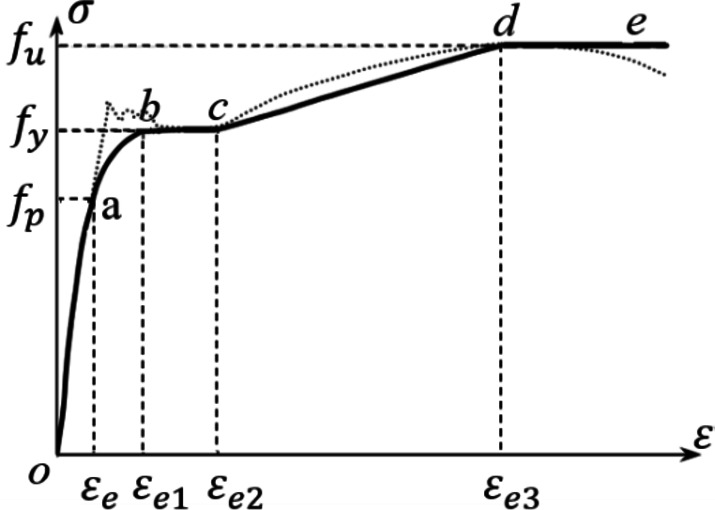
Stress–strain relationship curve of steel plate.

For the steel box, as shown in Fig. 10, the secondary-plastic-flow model from the literature^34^ was adopted.

1 $$\sigma = \left\{ \begin{gathered} E_s \varepsilon_s \, \varepsilon_s \leqslant \varepsilon_e \hfill \\ - A\varepsilon_s^2 + B\varepsilon_s + C \, \varepsilon_e < \varepsilon_s \leqslant \varepsilon_{e1} \hfill \\ f_y \, \varepsilon_{e1} < \varepsilon_s \leqslant \varepsilon_{e2} \hfill \\ f_y \left[ {{1} + {0}{\text{.6}}\frac{{\varepsilon_s - \varepsilon_{e2} }}{{\varepsilon_{e3} - \varepsilon_{e2} }}} \right] \, \varepsilon_{e2} < \varepsilon_s \leqslant \varepsilon_{e3} \hfill \\ 1.6f_y \, \varepsilon_s > \varepsilon_{e3} \hfill \\ \end{gathered} \right.$$

In the formula, the physical quantities are expressed as follows,

$$\varepsilon_e = 0.8f_y /E_s ,\varepsilon_{e1} = 1.5\varepsilon_e ,\varepsilon_{e2} = 10\varepsilon_{e1} ,\varepsilon_{e3} = 100\varepsilon_{e1} ,$$

$$A = 0.2f_y /\left( {\varepsilon_{e1} - \varepsilon_e } \right)^2 ,B = 2A\varepsilon_{e1} ,C = 0.8f_y + A\varepsilon_e^2 - B\varepsilon_e .$$

**Fig. 11:**
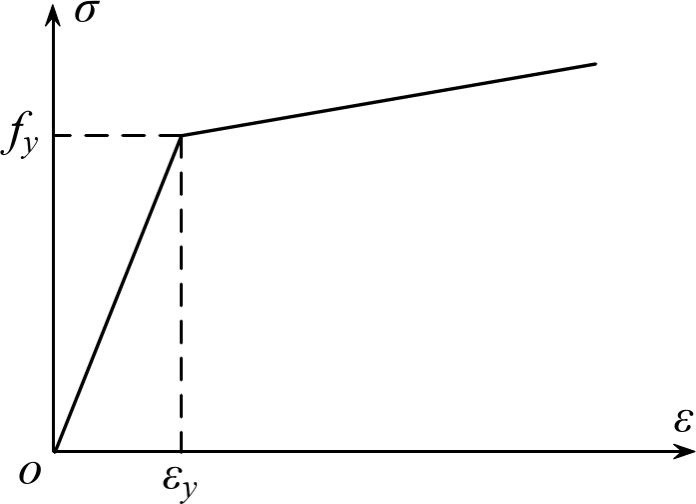
Stress–strain relationship curve of prestressed steel bar.

For the prestressed steel bar, as shown in Fig. 11, the ideal elastic–plastic bilinear model was adopted.For both, Poisson’s ratio was taken to be 0.3.

2 $$\sigma = \left\{ \begin{gathered} E_s \varepsilon_s \, \varepsilon_s \leqslant \varepsilon_e \hfill \\ 0.01E_S \left( {\varepsilon_s - \varepsilon_e } \right) + f_y \, \varepsilon_e < \varepsilon_s \hfill \\ \end{gathered} \right.$$

**Fig. 12:**
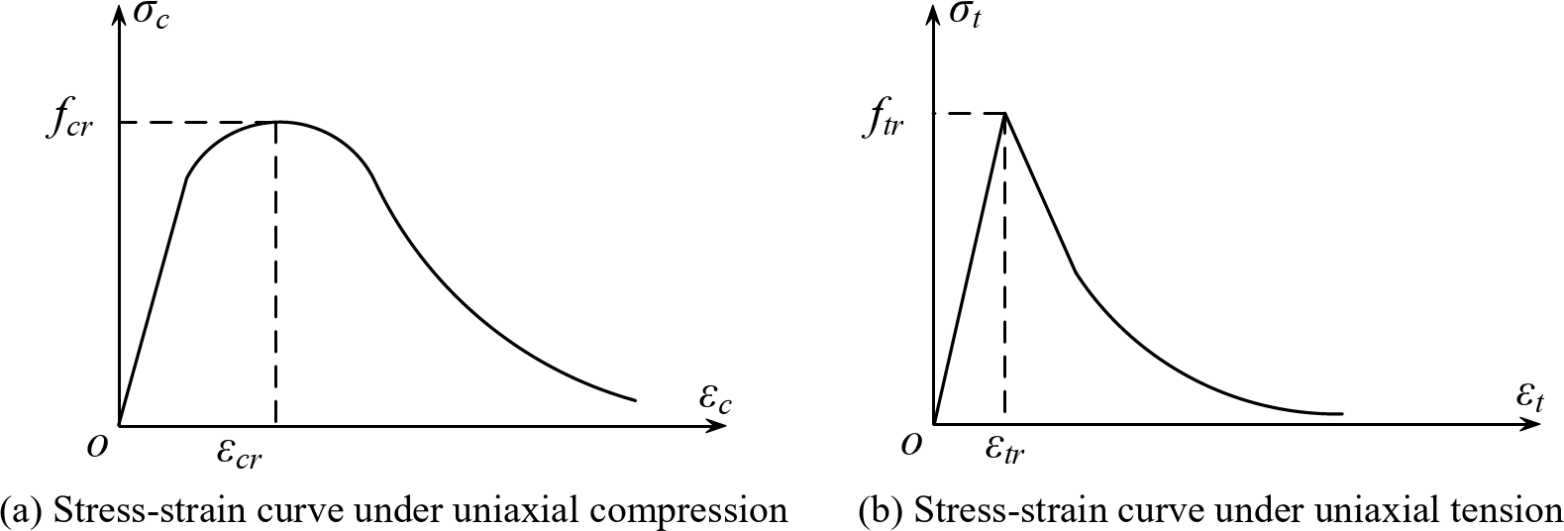
Stress–strain relationship curve of concrete.

The concrete model adopted was the plastic damage model provided by ABAQUS; Dilation Angle = 30, Eccentricity = 0.1, *f*_*b*0_/*f*_*c*0_=1.16, *k =* 0.6667, Viscosity Parameter = 0.0005. As shown in Fig. 12, the uniaxial tensile and compressive stress–strain curves of concrete were calculated according to the formula given by the Chinese standard^35^, with a Poisson’s ratio of 0.2. When concrete is subjected to uniaxial compression, it can be calculated using the following formula,

3 $$\sigma = \left( {1 - d_c } \right)E_c \varepsilon$$

4 $$d_c = \left\{ \begin{gathered} {1} - \frac{\rho_c n}{{n - {1} + x^n }} \, x \leqslant 1 \hfill \\ 1 - \frac{\rho_c }{{\alpha_c \left( {x - 1} \right)^2 + x}} \, x > 1 \hfill \\ \end{gathered} \right.$$

In the formula, the physical quantities are expressed as follows,

$$x = \frac{\varepsilon }{{\varepsilon_{cr} }},\rho_c = \frac{{f_{cr} }}{{E_c \varepsilon_{cr} }},n = \frac{{E_c \varepsilon_{cr} }}{{E_c \varepsilon_{cr} - f_{cr} }},\varepsilon_{cr} = \left( {700 + 172\sqrt {{f_{cr} }} } \right) \times 10^{ - 6} ,\alpha_c = 0.157f_{cr}^{0.785} - 0.905.$$

*α*_*c*_ is the parameter value of the descending segment of the uniaxial compressive stress–strain curve of the concrete; *d*_*c*_ is the damage evolution coefficient of concrete under uniaxial compression; *f*_*cr*_ is the representative value of the uniaxial compressive strength of concrete; *ε*_*cr*_ is the peak compressive strain of concrete corresponding to the compressive strength of concrete.

For uniaxial tension of concrete, the calculation is done according to the following formula,

5 $$\sigma = \left( {1 - d_t } \right)E_c \varepsilon$$

6 $$d_t = \left\{ \begin{gathered} {1} - \rho_t \left( {1.2 - 0.2x^5 } \right) \, x \leqslant 1 \hfill \\ 1 - \frac{\rho_t }{{\alpha_t \left( {x - 1} \right)^{1.7} + x}} \, x > 1 \hfill \\ \end{gathered} \right.$$

In the formula, the physical quantities are expressed as follows,

$$x = \frac{\varepsilon }{{\varepsilon_{tr} }},\rho_t = \frac{{f_{tr} }}{{E_c \varepsilon_{tr} }},\varepsilon_{tr} = f_{cr}^{0.54} \times 65 \times 10^{ - 6} ,\alpha_t = 0.312f_{tr}^2 .$$

*α*_*t*_ is the parameter value of the descending segment of the uniaxial tensile stress–strain curve of concrete; *d*_*t*_ is the damage evolution coefficient of concrete under uniaxial tension; *f*_*tr*_ is the representative value of the uniaxial tensile strength of concrete; *ε*_*tr*_ is the peak tensile strain of concrete corresponding to the compressive strength of concrete.

### Modelling process

**Fig. 13:**
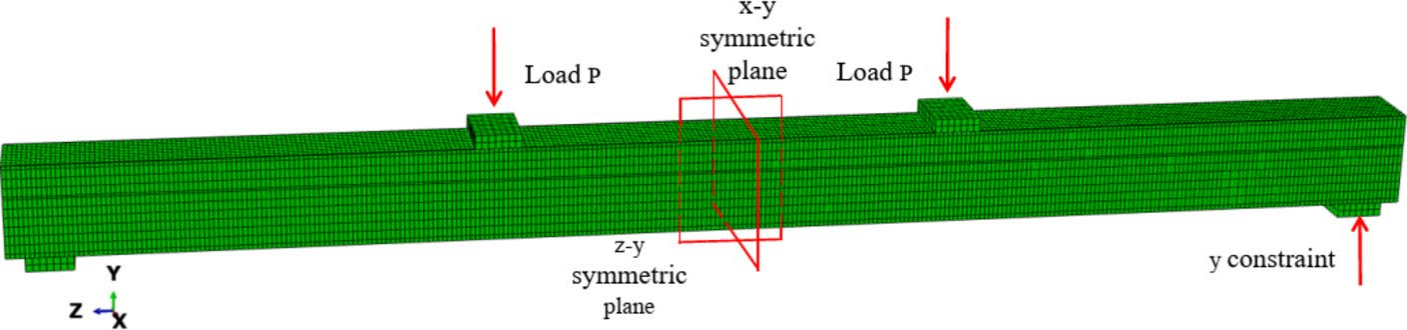
Finite element model.

The steel box and the concrete were represented by elements of type C3D8R, C3D8R elements are 8-node linear hexahedral elements that utilize reduced integration to prevent shear locking, which makes them suitable for solving nonlinear problems. Their advantages include resistance to shear locking under bending loads, accurate displacement solution results, and maintaining analysis precision even when the mesh experiences significant deformation. Whereas the prestressed reinforcement was represented by elements of type B31. B31 elements are one-dimensional beam elements used to model components where one dimension (length) is much larger than the other two, with stress being predominantly along the length. Prestressed reinforcement fits this characteristic well, and compared to higher-order elements, B31 elements can enhance computational efficiency while ensuring a certain level of calculation accuracy. The boundary conditions are shown in Figs. 1, 2 and 13, and 3 refer to the positive directions of the x, y, and z axes, respectively. The positive direction of the x-axis is perpendicular to the paper and faces outward, the positive direction of the y-axis is vertically upward, and the positive direction of the z-axis is horizontally to the left. x-y symmetric plane (U3 = UR1 = UR2 = 0), y-z symmetric plane (U1 = UR2 = UR3 = 0), and support point (U2 = 0). The load was applied by displacement. Motion coupling constraints (coupling) are applied between the ends of the reinforcement bars and the corresponding areas of the steel box end plates. Friction constraints are used between the steel box and the concrete, which include two parts: normal action and tangential action between the contact surfaces. The normal action employs hard contact (“Hard” Contact), which means that when the distance between the two surfaces is zero, pressure is generated between the contact surfaces without interpenetration; when the distance between the two surfaces is greater than zero, the contact surfaces separate, and the normal constraint disappears. Compared to the actual situation, in the test, the normal constraint between the steel box and the concrete can withstand tensile stress, but this difference does not affect the analysis of the load-bearing capacity. The tangential action uses a penalty function with a friction coefficient set at 0.4. General contact is set between the reinforcement bars and the transverse diaphragms, ensuring that after contact, the reinforcement bars can follow the deformation without penetrating the diaphragms and concrete. Because the structure and the load were symmetrical, the 1/4 model was used in the calculation. The prestress application was simulated using the cooling method. The initial temperature field of the prestressed steel bar was 0 °C, and the expansion coefficient of the steel bar was set at α = 1.2 × 10^− 5^. The relationship between the required prestress *F* and the temperature change was calculated from *T* = *F*/*E*_*s*_ × α × *A*_*p*_, where *E*_*s*_ and *A*_*p*_ are the elastic modulus and total area of the steel bar, respectively.

### Model verification

**Fig. 14:**
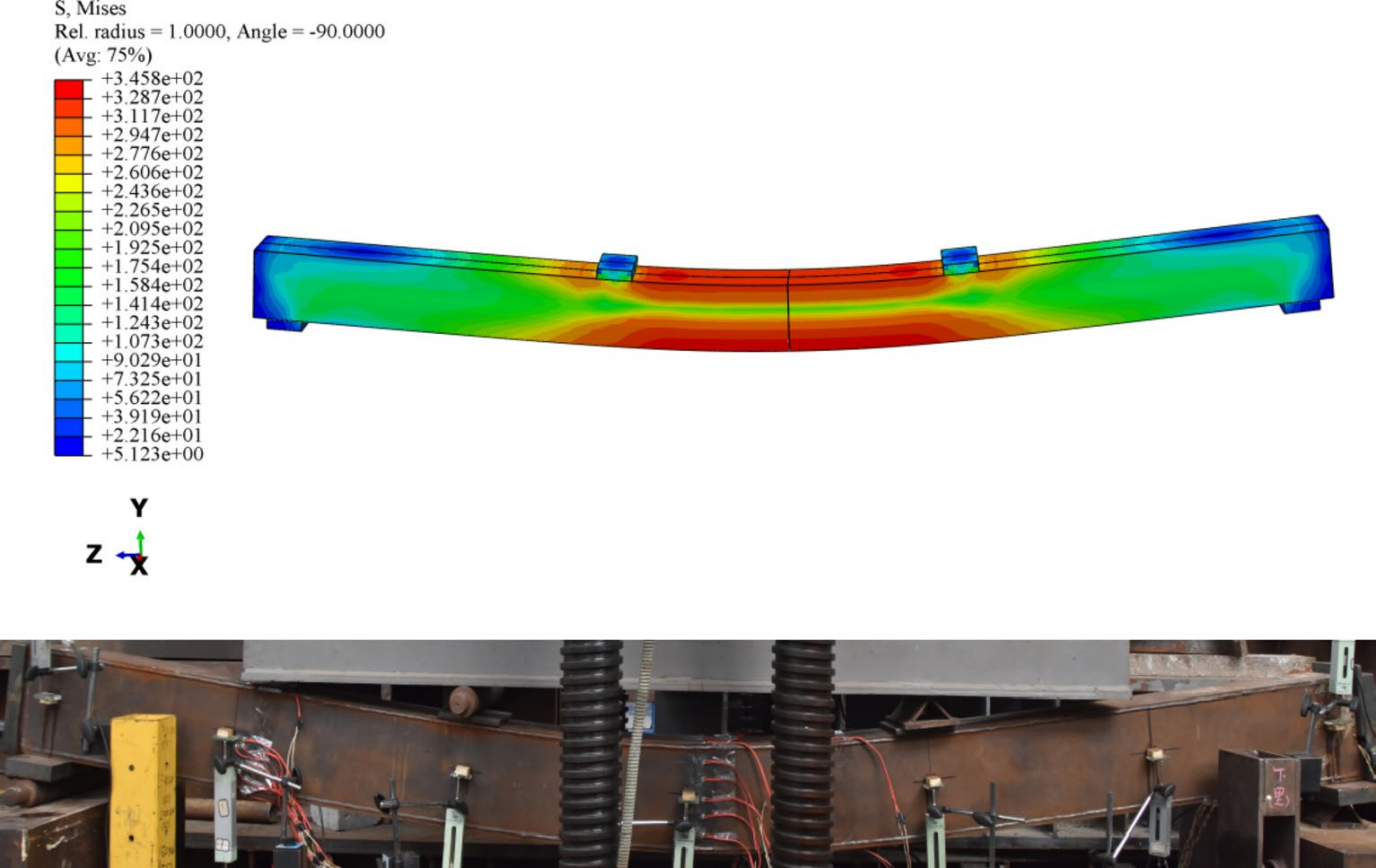
Deformation mode.

Taking specimen BR2-D as an example (Fig. 14), the deformation mode obtained using the FEM was consistent with the experimental results. The moment–strain and moment–deflection curves from the FEM analysis are shown in Figs. 7 and 8. The overall trends of the FEM analysis curves were consistent with those of the test curves. In the later stage of loading, especially for components with high filling ratios, the simulated moment–deflection curve was slightly lower than that in the experimental. This may be because the concrete was in a three-dimensional compression state in the later stage of loading, and the steel box had a hoop effect on the concrete; under these circumstances, the FEM model of the concrete constitutive process may be unrealistic. According to Table 2, the average values of the ratios of the FEM to the test results for the cracking moment, yield moment, and ultimate moment were 0.979, 1.018, and 0.995, respectively, with mean square errors of 0.042, 0.045, and 0.022. The above results indicate that the FEM can simulate the mechanical properties of PURCFSB beams.

## Numerical results and discussion

### Effects of different concrete filling ratios

To analyse the influence of the concrete filling ratio on the bearing capacity of a PURCFSB beam, the single-variable method was used to compare the results when the filling ratio of the design model varied from 0 to 100%. The geometric parameters, concrete cracking moment, yield moment, and ultimate moment of the model are shown in Table 3. When the tensile edge strain of the beam reached 0.01 and there was no load-drop section, the bending moment was the ultimate bending moment. When there was a load-drop section, the maximum bending moment before the strain reached 0.01 was the ultimate bending moment. The outer contour size of the model, the yield strengths of the steel box and the prestressed steel bar, and the concrete strength were the same in all cases; the prestress level was 0.5.

**Fig. 15:**
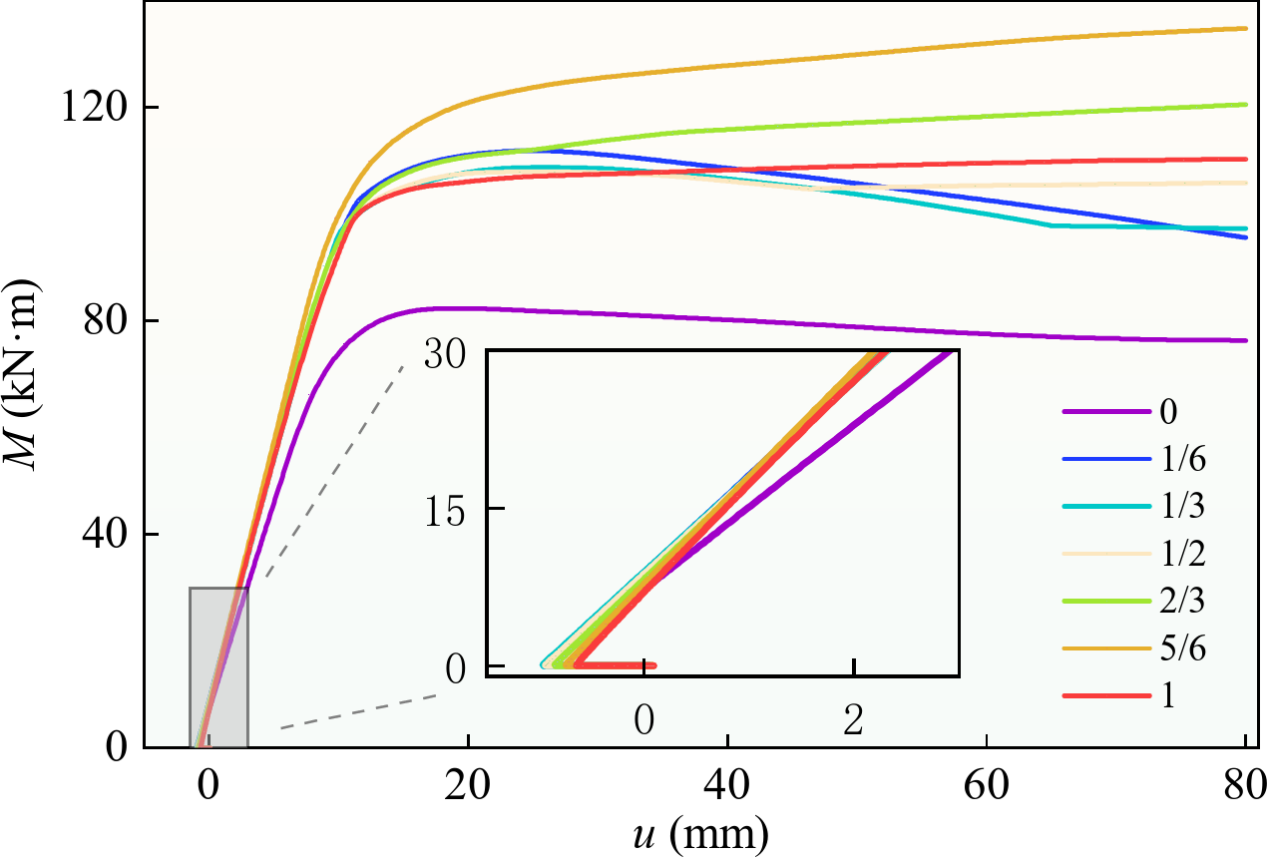
Influence of different filling rate on beam.

The bending moment–Displacement curve of the model is shown in Fig. 15. In the process of applying a load, the bending moment increased linearly with increasing deflection in the initial stage, and the initial stiffness increased with increasing filling ratio. As the concrete compression zone gradually expanded, the compressive stress of the concrete increased continuously. The yielding of the steel box caused the concrete to lose the restraint effect, and the component quickly reached the ultimate capacity. According to Table 3; Fig. 15, when the concrete filling ratio was less than 2/3, no concrete cracking occurred in the normal use stage; when the filling ratio was less than 1/2, the concrete beams never cracked during the whole process of bending. Compared with those of the unequal-walled steel-box beams, the yield moment of the PURCFSB beam increased by 30.3–45.1%, and the ultimate moment increased by 30.9–60.8%. Compared with the unequal-walled CFST beam, the yield moment increased by 19.7%, and the ultimate moment increased by 21.0%. When the filling ratio of the concrete was greater than or equal to 1/2, the structure did not crack before yielding. When the filling ratio was between 1/6 and 1/2, the ultimate moment did not change significantly, the distance between the diaphragm and the neutral axis decreased, and the bending moment decreased when the stress was the same. The increased concrete was in the compression zone, which improved the bearing capacity. The ultimate moment increased between 1/2 and 5/6; the distance between the position of the diaphragm and the neutral axis increased, and the bending moment increased when the stress was the same. The ultimate moment of the fully filled beam was almost the same as that with a filling ratio of 1/2 because the increased concrete was in the tensile zone, the bearing capacity was poor, and the beam had no diaphragm when the filling ratio was 1.

**Table 3:** Specimen parameters of different concrete filling rate. *t*_*w*_ is the thickness of web and diaphragm, *f*_*y*_ is the yield strength of stell box.

Specimen	a	t_1_ (mm)	t_2_ (mm)	t_w_ (mm)	L (mm)	b (mm)	h (mm)	f_y_ (MPa)	f_py_ (MPa)	f_cu_ (MPa)	M_cr_ (KN·m)	M_y_ (KN·m)	M_u_ (KN·m)
RB1	0	8	10	5	3000	200	150	235	450	60	/	66.3	82.5
RB2	1/6	8	10	5	3000	200	150	235	450	60	/	86.4	112.1
RB3	1/3	8	10	5	3000	200	150	235	450	60	/	94.8	108.9
RB4	1/2	8	10	5	3000	200	150	235	450	60	89.3	88.7	108.0
RB5	2/3	8	10	5	3000	200	150	235	450	60	46.5	90.2	118.4
RB6	5/6	8	10	5	3000	200	150	235	450	60	39.8	96.2	132.7
RB7	1	8	10	5	3000	200	150	235	450	60	22.1	80.4	109.7

### Effects of the prestress levels

To study the influence of prestress on bending capacity, eight beams with the same outline size, steel-box and prestressed-steel-bar yield strengths, and concrete strength were simulated; according to the test results, the load bearing performance is better when the filling ratio is 1/2, so the filling ratio was 1/2 in every case. One beam did not contain prestressed steel bar; the prestress levels *δ* of the other beams were 0, 1/6, 1/3, 1/2, 2/3, 5/6, and 1, The model parameters and ultimate moment are shown in Table 4.

**Fig. 16:**
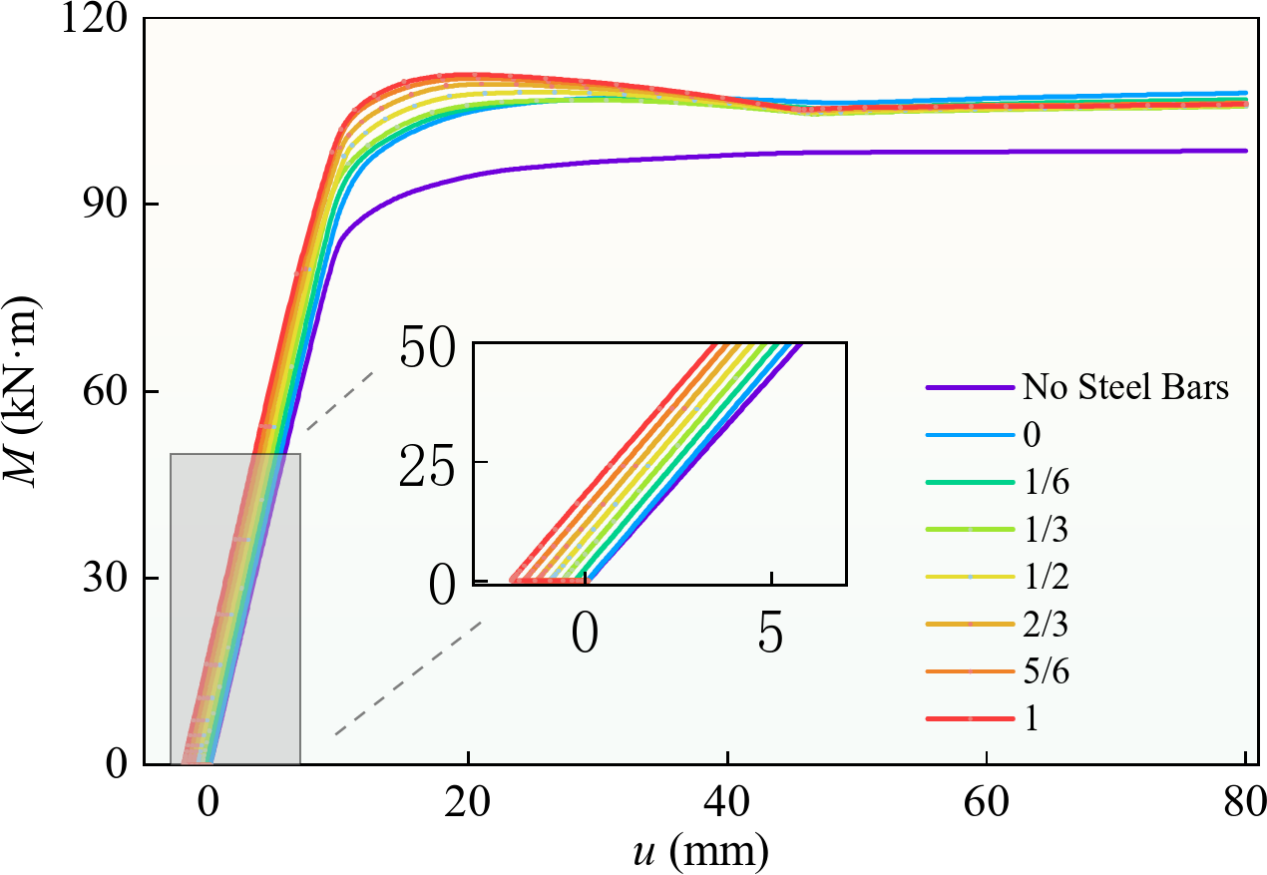
Influence of prestress on moment–deflection curve.

Figure 16 shows the influence of different prestress levels on the bending moment–deflection curve. Figure 16; Table 4 show that compared with that of the beam without a prestressed steel bar (BR1), the initial stiffness (*K* in Table 4 ) of the prestressed beams (BR2-8) was improved, the maximum yield bending moment increased by 19.5%, and the maximum limiting bending moment increased by 17.1%. These results indicate that prestress can improve the initial stiffness, yield bending moment, and ultimate bending moment of a structure. The initial cambers of different prestressed horizontal beams are different, and the cambers increase with increasing prestress level.

The bending moment–deflection curves corresponding to different prestress levels were initially almost parallel. Compared with the 0 and 1 prestress levels, the prestress increased the yield moment by 9.1% and the ultimate moment by only 3.5%, indicating that the prestress level had little effect on the initial stiffness and ultimate moment. In practical engineering applications, the prestress should be chosen in accordance with the design specification, and so that the upwarp deflection does not exceed 1/1000 of the span, and the prestress is 50–85% of the yield strength of the prestressed steel bar.

**Table 4:** Specimen parameters of different prestress levels.

Specimen	δ	t_1_ (mm)	t_2_ (mm)	t_w_ (mm)	L (mm)	b (mm)	h (mm)	f_y_ (MPa)	f_py_ (MPa)	f_cu_ (MPa)	M_y_ (KN·m)	M_u_ (KN·m)	K (10^3^KN·m^2^)
BR1	/	8	10	5	3000	200	150	235	/	60	82.4	94.7	8.1
BR2	0	8	10	5	3000	200	150	235	450	60	90.3	107.2	8.4
BR3	1/6	8	10	5	3000	200	150	235	450	60	91.9	107.0	8.5
BR4	1/3	8	10	5	3000	200	150	235	450	60	92.8	106.8	8.5
BR5	1/2	8	10	5	3000	200	150	235	450	60	93.5	108.1	8.4
BR6	2/3	8	10	5	3000	200	150	235	450	60	95.1	109.4	8.4
BR7	5/6	8	10	5	3000	200	150	235	450	60	97.2	110.3	8.4
BR8	1	8	10	5	3000	200	150	235	450	60	98.5	110.9	8.4

## Conclusion

The PURCFSB beam was investigated experimentally and numerically. The following results were obtained:

(1) According to the test results, all specimens yielded when the displacement reached about 20 mm, and until the displacement reached the test termination condition of 100 mm, except for when the concrete filling ratio was 0 and 1/3 (which caused local buckling), there was no load drop section, proving that the PURCFSB beams have good ductility.

(2) When the beam filling ratio was less than 1/3, local buckling might have occurred in the later stage of loading. When the filling ratio was less than 1/2, the concrete cracking of the specimen could be avoided. The yield moment and ultimate moment of the PURCFSB beams with proper filling ratios (1/3–1/2) and prestress levels (50–85%) were obviously greater than those of the unequal-walled steel-box and CFST beams.

(3) According to the results from the finite element numerical simulation and experimental tests, compared to beams without prestressed reinforcement, the addition of prestressed reinforcement can improve the initial stiffness, yield moment, and ultimate moment of unequal-walled rectangular concrete-filled steel-box beams. For beams that have been provided with prestressed reinforcement, the magnitude of the prestress level significantly affects the cracking moment; specimens with larger concrete tensile zones exhibit this effect more prominently. The level of prestress has a minimal impact on the initial stiffness and ultimate bending moment of the beam, but it does enhance the yield bending moment.

(4) The moment–deflection curve of the PURCFSB beam initially had the form of an inverted arch, which could prevent a large-span structure from exceeding its safe working range as a result of excessive deformation. The combined action of prestress and partial filling with concrete not only improved the structure’s bending capacity, but also reduced its weight.

## Data Availability

The datasets generated and/or analysed during the current study are not publicly available due to our laboratory confidentiality agreement but are available from the corresponding author on reasonable request.

## List of Symbols

*L*, *L*_0_: Total length and effective length of specimens, respectively
*h*, *b*: Outer width and height of section, respectively
*t*_1_,* t*_2_,* t*_*w*__1_,* t*_*w*__2_: Thickness of upper flange plate, lower flange plate, web, and diaphragm, respectively
*f*_1_, *f*_2_: Yield strength of upper and lower flange plates, respectively
*f*_*w*_: Yield strength of web and diaphragm
*f*_*py*_, *f*_*p*__*u*_: Yield strength and tensile strength of prestressed steel bar
*f*_*cu*_: Compression strength of concrete
*a*: Concrete-filled ratio
*δ*: Ratio of prestressing to yield strength of Q235 steel
*M*_*y*__1_, *M*_*y*__2_: Yield moment of upper and lower flange plates, respectively
*M*_*u*_: Ultimate moment
*M*_*cr*_: Concrete crack moment
*E*_*s*_: Elastic modulus of steel bar
*A*_*p*_: Total area of steel bar
